# Supplementary material for: Social implications of the 30×30 global conservation target
Source: Nat Commun. 2026 May 12;17:4067. doi: 10.1038/s41467-026-71860-8 (PMC13168258; doi:10.1038/s41467-026-71860-8)
Supplement: Supplementary file 1 — Supplementary Information [file 41467_2026_71860_MOESM1_ESM.pdf]

Supplementary Information for:

Social implications of the 30x30 global conservation target

Javier Fajardo*[1,2], Heather C. Bingham[3], Dan Brockington[2,4,5], Rebecca Chaplin-Kramer[6], James A. Fitzsimons[7,8,9], Forrest Fleischman[10], Alain Frechette[11], Rachael D. Garrett[1], Carolina Hazin[12], Tobias Kuemmerle[13,14], Janeth Lessmann[3], Milagre O. F. Nuvunga[15], Brian O'Donnell[16], Fred Onyai[17], Ruth Pinto[18], Marion Pfeifer[19], Rose Pritchard[20], Casey M. Ryan[21], Priya Shyamsundar[22], Josefa Cariño Tauli[23,24], David Mwesigye Tumusiime[25], Jasmin Upton[3], Gary R. Watmough[21], Julie G. Zaehringer[26,27], Chris Sandbrook*[1]

[1]Department of Geography and Conservation Research Institute, University of Cambridge, Cambridge, UK
[2]Institut de Ciència i Tecnologia Ambientals de la Universitat Autònoma de Barcelona (ICTA-UAB), Cerdanyola del Vallès, Spain
[3]UN Environment Programme World Conservation Monitoring Centre, Cambridge, UK
[4]Department of Private Law, Universitat Autònoma de Barcelona, Cerdanyola del Vallès, Spain
[5]ICREA, Barcelona, Spain
[6]WWF, San Francisco, USA
[7]The Nature Conservancy, Carlton, VIC, Australia
[8]School of Life and Environmental Sciences, Deakin University, Burwood, VIC, Australia
[9]School of Law, University of Tasmania, Sandy Bay, TAS, Australia
[10]Department of Forest Resources, University of Minnesota, St Paul, MN, USA
[11]Rights and Resources Initiative, Washington, DC, USA
[12]The Nature Conservancy, London, UK
[13]Geography Department, Humboldt-University Berlin, Berlin, Germany
[14]Integrative Research Institute on Transformations in Human-Environment Systems (IRI THESys), Humboldt-University Berlin, Berlin, Germany
[15]Micaia Foundation, Chimoio, Mozambique
[16]Campaign for Nature, Durango, CO, USA
[17]Private Consultant, Kampala, Uganda
[18]International Institute for Environment and Development, London, UK
[19]SNES, Newcastle University, Newcastle Upon Tyne, UK
[20]Global Development Institute, University of Manchester, Manchester, UK
[21]University of Edinburgh, School of Geosciences, Edinburgh, UK
[22]The Nature Conservancy, Arlington, VA, USA
[23]Global Youth Biodiversity Network, WY, USA
[24]Partners for Indigenous Knowledge Philippines, Baguio City, Philippines
[25]Department of Environmental Management, Makerere University, Kampala, Uganda
[26]Wyss Academy for Nature, Bern, Switzerland
[27]Centre for Development and Environment and Institute of Geography, University of Bern, Bern, Switzerland

Corresponding author emails:
Javier Fajardo: fajardonjavier@gmail.com
Chris Sandbrook: cgs21@cam.ac.uk

Supplementary Information for:

Social implications of the 30x30 global conservation target

Table of Contents

Supplementary Discussion 3

1. Area in Target 3 scenario and neighbour areas 3
2. Overlap among Target 3 scenarios 4

Area in variations of the ITT scenario 9

3. Resident population projection in 2030 10
4. Sensitivity analyses: limitations to the global social datasets and methods 11

4.1 Introduction 11

4.2 Sensitivity analysis of population datasets 11

4.3 Sensitivity analysis of Target 3 scenario layer selection 12

4.4 Sensitivity analysis of local wealth conditions 16

4.5 Sensitivity analysis of potential impact on livelihoods 18

4.6 Sensitivity analysis of scenario neighbouring areas 19

Supplementary Tables 21

Supplementary Table 1 22

Supplementary Table 2 23

Supplementary Table 3 24

Supplementary Table 4 25

Supplementary Table 5 27

Supplementary Table 6 31

Supplementary References 32

Supplementary Discussion

1. Area in Target 3 scenario and neighbour areas

Target 3 scenarios, including current protected and conserved areas, cover 30% of the global terrestrial and inland water area (Supplementary Fig. 1a). The extent of adjacent areas (i.e., within 10 km of future protected and conserved areas) varies across scenarios. The Biodiversity-based scenario includes the largest total area within buffer zones, covering 54% of the global terrestrial and inland water area, while the Nature's Contributions to People (NCP)-based and Indigenous and traditional territories (ITT) scenarios each include approximately 50% within these adjacent zones.

At the continental scale (Supplementary Fig. 1b), both the extent of future areas in Target 3 scenarios and their neighbouring zones vary across continents. This variation reflects differences in both the current coverage of protected and conserved areas and the additional areas designated in each scenario. Current protected and conserved areas range from approximately 12% in Asia to 26% in South America. When scenarios' areas are considered, the smallest coverage is found in Europe under the ITT scenario (20%), while the largest is in South America under the NCP-based scenario (over 50%). Including neighbouring areas increases total coverage unevenly among continents. South America continues to include the highest overall coverage across scenarios, surpassing 70% in the NCP-based scenario and approaching this percentage in the Biodiversity-based scenario. In contrast, Europe, which has relatively small current coverage of protected and conserved areas and scenario-designated areas, ranks among the continents with the highest total coverage when buffers are included, with two scenarios exceeding 60%. This is due to the smaller and more numerous protected areas (PAs) in Europe, which result in a greater total boundary length and, consequently, a larger buffer area.

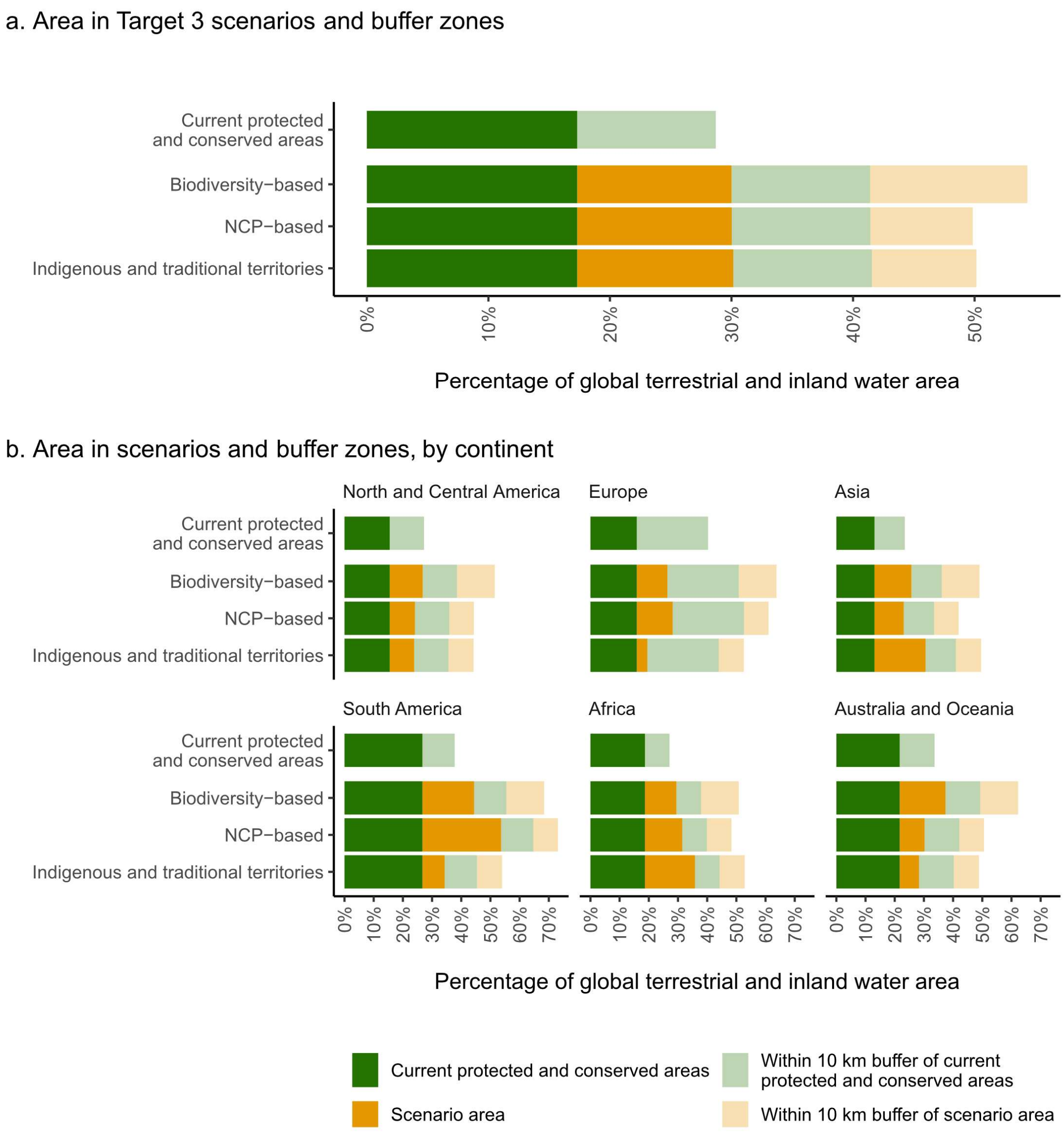

Supplementary Figure 1: Percentage of global terrestrial and inland water area in existing protected and conserved areas and Target 3 scenarios, and within 10 km distances of each, at the global scale (a) and by continent (b).

2. Overlap among Target 3 scenarios

We used the 'terra' R package to calculate the areas that were unique to each scenario and those that overlapped between them (Supplementary Fig. 2). We then generated Venn diagrams with the 'eulerr' package in R to visualise these overlaps, expressed either as percentages of the new nominal area of each scenario (i.e., 12.8% of the global

area; the standardised target area assigned to each scenario, excluding current protected and conserved areas; Supplementary Fig. 3a) or relative to the global land area (Supplementary Fig. 3b). As a result of expressing overlaps as percentages of the new nominal area in each scenario (Supplementary Fig. 3a), the summed proportions for a given scenario may not add exactly to 100%. This small deviation reflects the minor differences in total scenario areas but ensures direct comparability among them.

The overlap among the Target 3 scenarios is relatively limited. Geographically, areas of scenario overlap concentrate in tropical regions (Supplementary Fig. 2). The largest overlap occurs between the Biodiversity-based and NCP-based scenarios, with 22.8% of their newly proposed areas coinciding (Supplementary Fig. 3a), representing 2.9% of the global terrestrial and inland water area (Supplementary Fig. 3b). In contrast, the overlap between the NCP-based and Indigenous and traditional territories scenarios is smaller, at 11.8% (1.5% of the global area). The overlap between Biodiversity-based and the Indigenous and traditional territories scenarios is the lowest of the pairwise

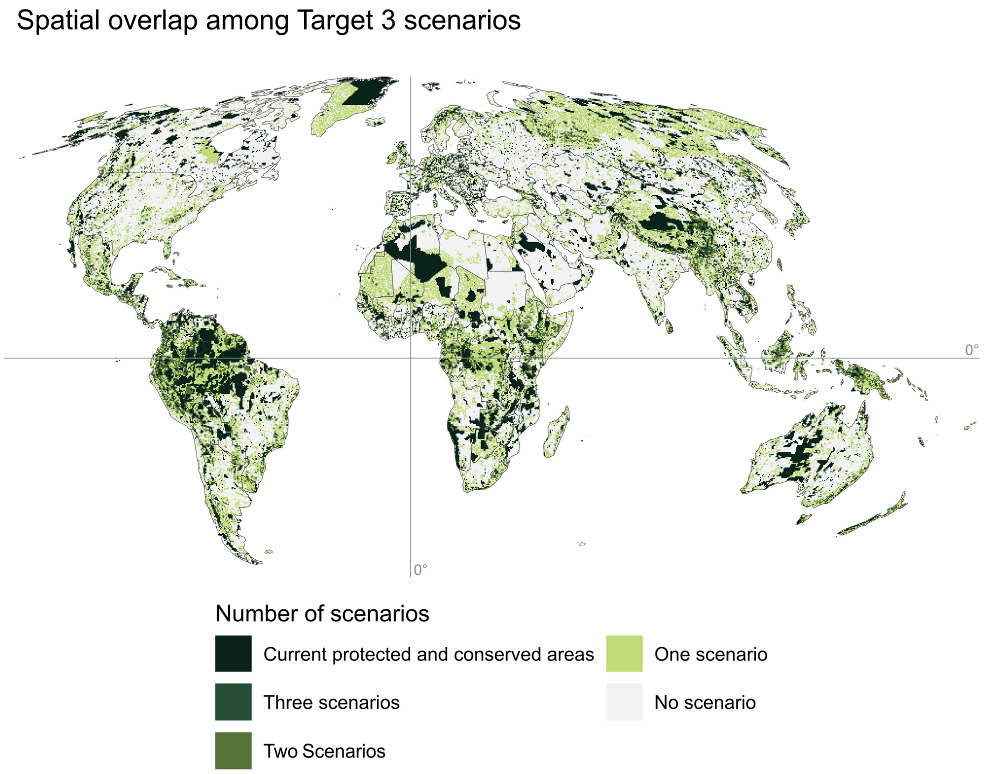

Supplementary Figure 2: Spatial overlap among the three Target 3 scenarios assessed. Colours from light to dark green indicate the number of scenarios in which a grid cell is included. Current protected and conserved areas are included in all scenarios by definition.

overlaps, at 7.9% (1.0% of the global area). Areas shared by all three scenarios constitute only a small fraction – 4.6% of the new area designated by the scenarios – covering just 0.6% of the global terrestrial and inland water area (in addition to 17.2% corresponding to existing protected and conserved areas). A significant portion of the Earth's land surface, 51.6%, is not included in any of the scenarios. Of the 48.4% of land included in at least one scenario, 17.2% is part of existing protected and conserved areas, 25.2% is covered exclusively by one scenario, 5.4% is shared by two scenarios, and 0.6% is common to all three.

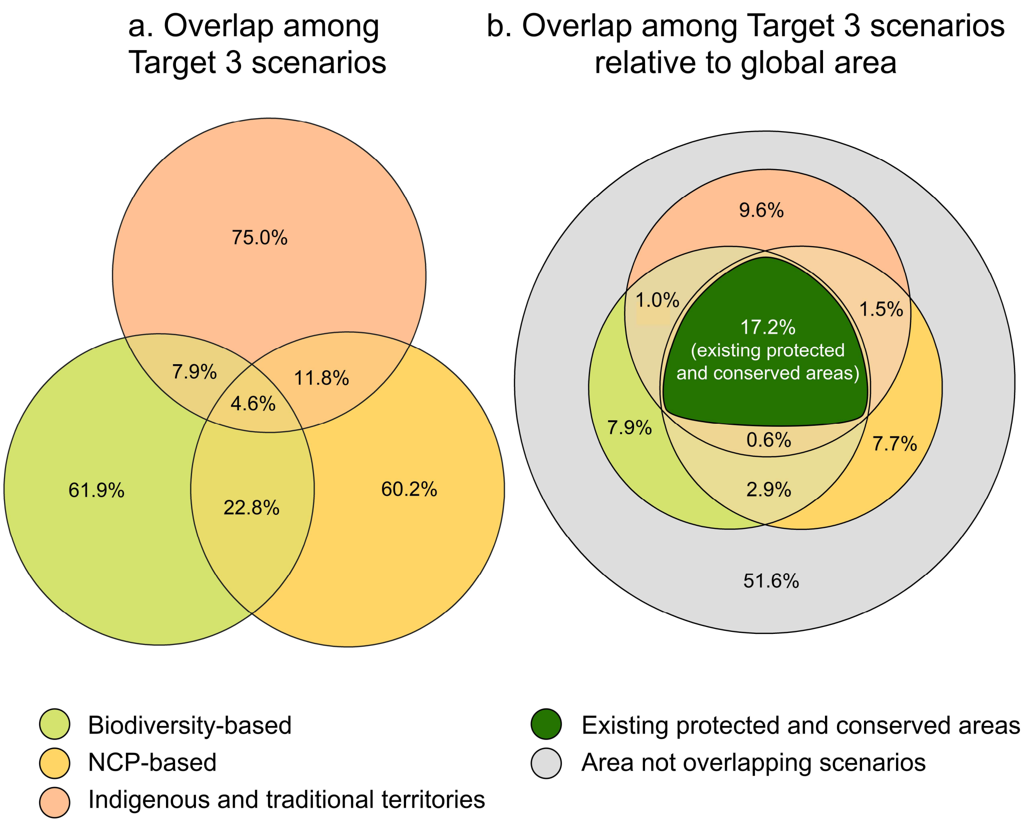

Supplementary Figure 3: Venn plots showing overlap among Target 3 scenarios. (a) Percentage overlap between scenarios, showing the proportion of the new nominal area of scenarios, excluding current protected and conserved areas, that overlaps with the others. (b) Percentage of global terrestrial and inland water areas covered by the overlapping regions of the scenarios, indicating the extent of the global area represented by scenarios and their overlaps. The green area at the centre corresponds to existing protected and conserved areas, which are common to all three scenarios, while the outer portion of the central area represents additional overlap among the three scenarios outside these areas. One randomly chosen ITT scenario layer was used to assess the overlap of the ITT-approach map with other scenarios.

We also estimated the socioeconomic profiles of populations residing in areas where the scenarios overlap, following the same procedure described in the main text (Fig. 2, main document). Here, we calculated total population, development status according to Human Development Index (HDI) groups, and livelihoods – specifically the share of population engaged in wild harvesting (for tropical areas only), and the extent of farm and livestock rangeland areas (Supplementary Fig. 4).

The largest total population occurs in the overlap area between the Biodiversity-based and NCP-based scenarios, encompassing 211 million people, well above the NCP–ITT (28.4 million), Biodiversity–ITT (14.6 million), and Biodiversity-NCP-ITT (14 million). Although these differences partly reflect the varying extent of overlaps (22.8%, 11.8%, 7.9%, and 4.6% of total new scenario area, respectively), population density (people per 1% of scenario area) is also higher in the Biodiversity–NCP overlap (9.25 million per 1%) than in any overlap involving the ITT scenario (1.85, 2.41, and 3.04 million per 1% for the NCP–ITT, Biodiversity–ITT, and Biodiversity-NCP-ITT overlaps, respectively). This indicates that the Biodiversity–NCP overlap occurs in more densely populated regions, whereas overlaps involving ITT are more sparsely inhabited. Nonetheless, compared to the new area in each scenario, overlap areas are less densely populated than the Biodiversity-based scenario as a whole, comparable to the NCP-based scenario, and denser than the ITT scenario.

Overall, socioeconomic profiles in overlap areas tend to represent intermediate conditions between the individual scenarios they connect. The Biodiversity–NCP overlap hosts a more socioeconomically diverse population, with smaller shares of low-HDI people and greater representation of high- and very-high-HDI groups compared to overlaps involving the ITT scenario. In contrast, the Biodiversity–ITT, NCP–ITT, and Biodiversity-NCP-ITT overlaps include smaller total populations but higher proportions of low- and medium-HDI groups, reflecting overall lower development status.

Livelihood patterns also differ among overlap areas. Populations in the Biodiversity–NCP overlap engage less on wild harvesting. About half of the area in this overlap includes livestock, whereas the presence of farm area (both small and large farms) is larger than in other scenario overlaps. Contrastingly, overlaps involving the ITT scenario, including the overlap among the three scenarios, show very large presence of

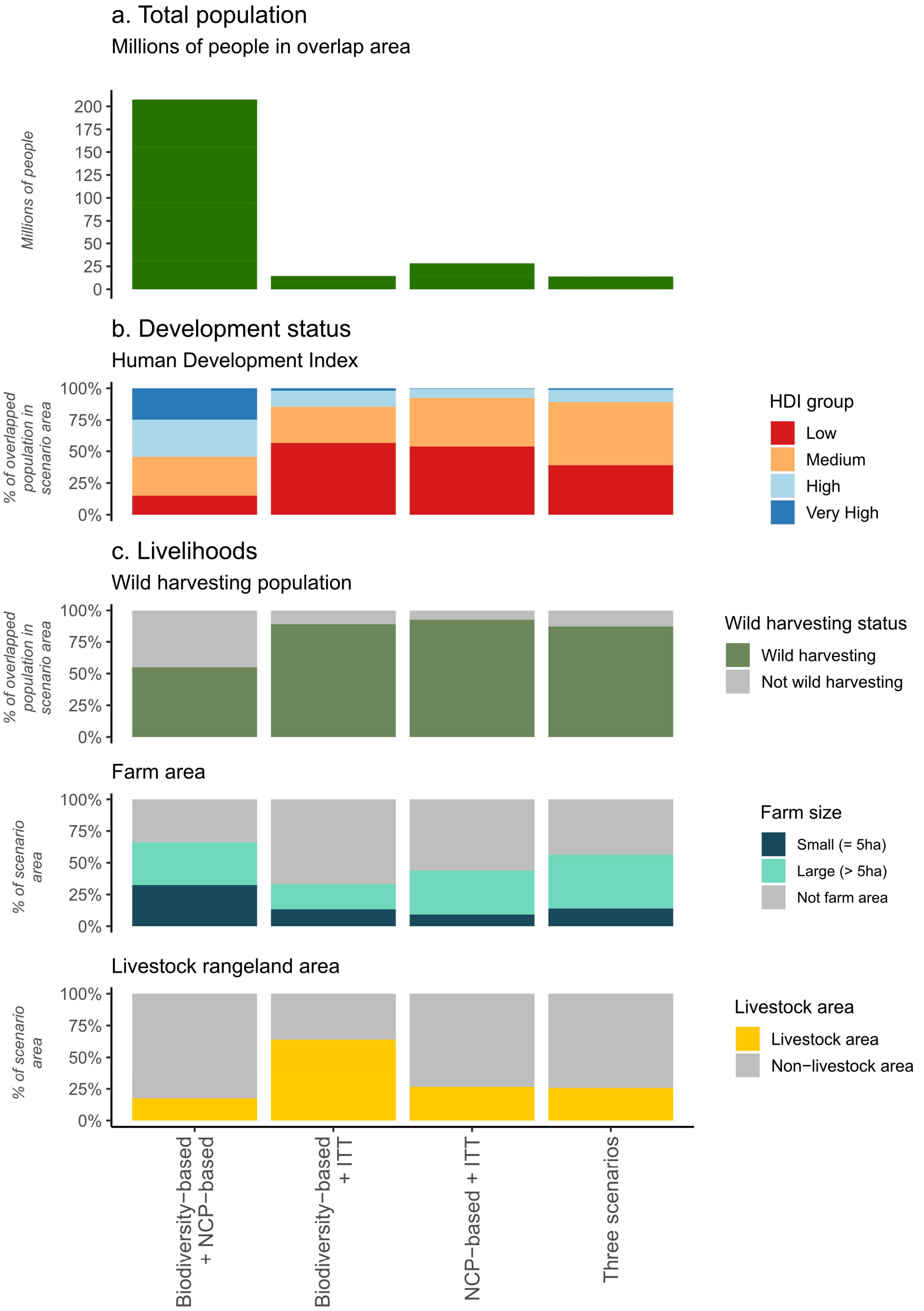

Supplementary Figure 4: Population and socioeconomic characteristics of overlap areas among Target 3 scenarios. (a) Total resident population in overlap areas. (b) Human Development Index (HDI) of populations, expressed as the percentage of people in each HDI group. (c) Livelihoods of populations, shown as the percentage of population or area by: wild harvesting status (tropical population), farm area – disaggregated into small farms (≤ 5 ha), large farms (> 5 ha), and non-farmed area – and livestock rangeland area. HDI thresholds: Low < 0.550; Medium 0.550–0.699; High 0.700–0.799; Very High > 0.800.

wild harvesting and less farm area. Presence of livestock area is the largest in the Biodiversity–ITT overlap area.

Area in variations of the ITT scenario

The methodology used to generate the ITT scenario produced a map covering 36% of global terrestrial and inland water areas. To adjust the coverage to a comparable Target 3 scenario of 30%, we introduced a step in which grid cells were randomly removed. To assess uncertainty arising from this random removal, the process was repeated 100 times, producing 100 distinct maps. Supplementary Fig. 5 presents a map showing the number of times each grid cell was selected across the 100 replicates. The original 25 km² resolution layer was upscaled for display to a 1°x1° degree resolution map to avoid displaying sensitive boundaries associated with Indigenous and Community Conserved Areas (ICCAs) and Indigenous Peoples and custodian communities' territories. The main results for this scenario, including population counts, population within HDI profiles, and livelihoods reported in the manuscript, reflect the mean values across the 100 replicates. Variability across replicates was minimal, with standard deviation values for all results provided in Supplementary Tables 1, 2, and 3.

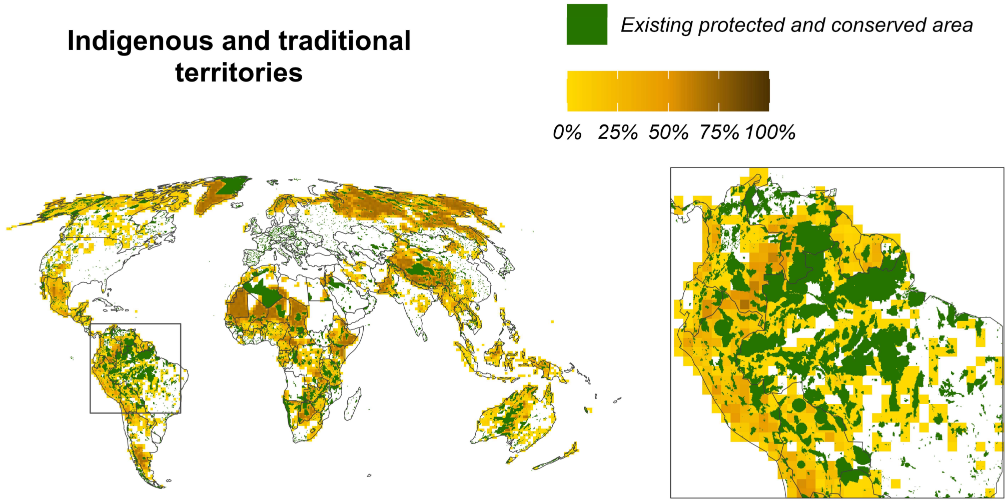

Supplementary Figure 5: Spatial distribution of areas included in the ITT scenario. The map displays the proportion of each 1°x1° degree grid-cell covered by areas in one of the 100 replicas of the scenario, aggregated from the original 25 km² resolution data. Colour from yellow to dark brown represents the percentage of area in the large grid cells, with darker colours indicating higher percentages. The map is presented in this aggregated resolution to preserve sensitive data related to Indigenous people and custodian communities territories used in building the scenario.

3. Resident population projection in 2030

Projected population trends towards 2030 using GHS-POP data indicate that the population living in existing protected and conserved areas and areas in Target 3 scenarios is expected to increase.

The largest increases compared to present population levels are in the Biodiversity-based scenario, with 209 million more people projected to inhabit those areas, a 11% increase with respect to the population in 2023 (Supplementary Fig. 6). This scenario is followed by the NCP-based, where the projected increase is of approximately 70 million people (a 11% increase compared to 2023). Lastly, the Indigenous and traditional territories is the scenario with the shortest projected population increase (27.5 million people), although it also corresponds to the highest proportional increase (almost a 30 % increase). For comparison, the population in existing protected and conserved areas is projected to increase by almost 36 million, which would represent a growth of 9% from 2023 values. The projected increase of resident population in scenarios seems therefore roughly proportional to global population growth trends for all scenarios except for the Indigenous and traditional territories scenario, where the rate is three times higher.

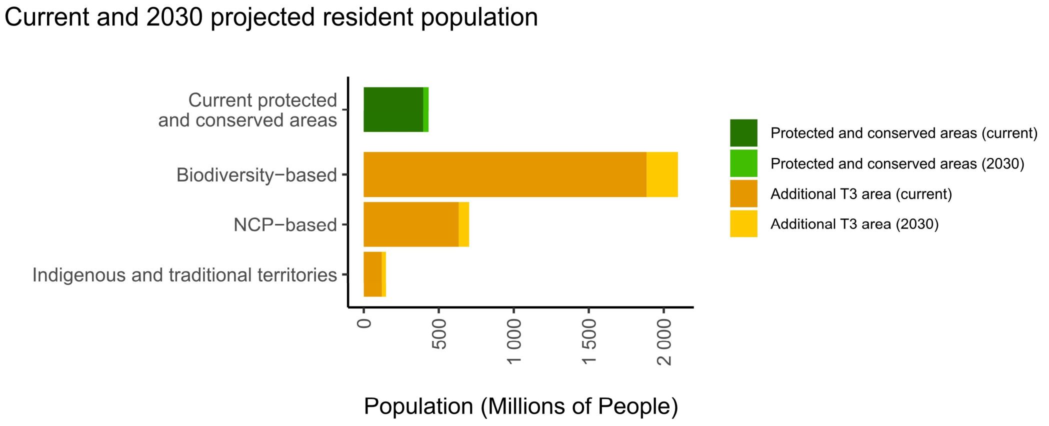

Supplementary Figure 6: Current and projected 2030 population in existing protected and conserved areas, and new areas under Target 3 scenarios.

4. Sensitivity analyses: limitations to the global social datasets and methods

4.1 Introduction

Our findings are contingent on the quality of the social datasets that we used as well as dependent on the location of areas highlighted by Target 3 scenarios.

In this section, we explore and discuss implications of the social data selected for analyses and of the layers used to build scenarios. The section provides a sensitivity analysis for the analyses by comparing results from the chosen data against alternatives.

4.2 Sensitivity analysis of population datasets

We evaluated how estimates of total population within the areas of each scenario varied when using population datasets alternative to the Global Human Settlement Population dataset[1] (GHS-POP): WorldPop[2] (constrained, unconstrained and UN adjusted-unconstrained projections), Gridded Population of the World[3] (GPWv4) and Landscan[4]. Variability across datasets was generally small and did not alter the ranking of scenarios in terms of the total population affected (Supplementary Fig. 7). The Biodiversity-based scenario consistently involved the largest potential resident population, ranging from 1.89 billion (GHS-POP) to 2.08 billion (WorldPop constrained). The NCP-based scenario affected an estimated 633 to 934 million people (GHS-POP and GPWv4, respectively), while the ITT scenario consistently involved the smallest total resident population, ranging from 121 to 203 million (GHS-POP and GPWv4). These figures are in addition to the 396 to 595 million people residing in current protected and conserved areas. Among the datasets, GHS-POP consistently produced the lowest estimates for existing PAs, OECMs, and all scenarios, whereas WorldPop constrained and GPWv4 tended to yield the highest estimates.

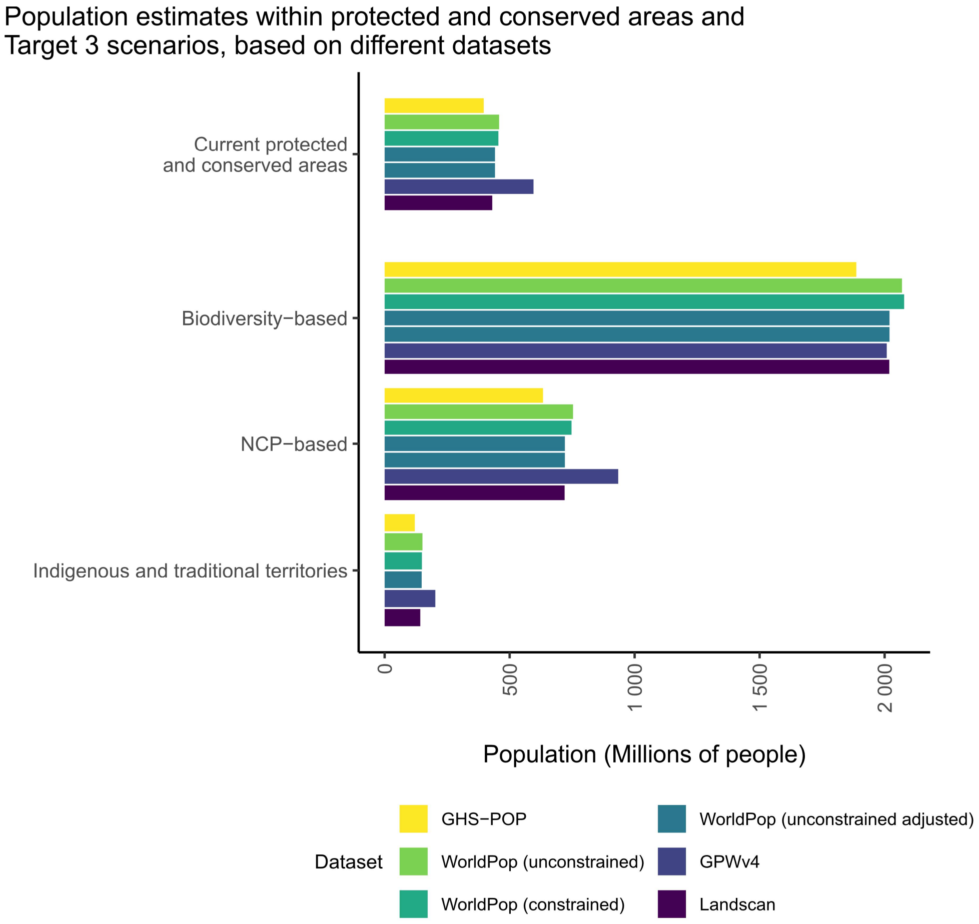

Supplementary Figure 7: Population in 2020 in current PAs and OECMs and Target 3 scenarios according to four global datasets: GHS-POP, WorldPop, GPWv4 and LandScan.

4.3 Sensitivity analysis of Target 3 scenario layer selection

What areas will be designated as protected or conserved under Target 3 is uncertain. Our scenarios represent three widely considered approaches – Biodiversity-, NCP-based, and ITT – and are designed to highlight areas aligning with the KMGBF criteria. However, spatial representations of these approaches can vary, for instance, due to dataset and method choices. We conducted sensitivity analyses to evaluate how alternative datasets and methods affect the results under each scenario class.

Across all scenario classes, total population counts, HDI profiles, and livelihood characteristics of potentially affected populations were generally consistent when

comparing results generated with alternative datasets (Supplementary Fig. 8). While specific values vary depending on the dataset used, the overarching conclusion is that distinct social implications arise from different approaches to determining future protected and conserved areas, making it critical to consider these implications during implementation.

Results for variations of scenarios under the Biodiversity-based scenario were highly consistent across evaluated variables. Variations introduced by Shen et al.'s[5] prioritisation using different ecoregion coverage targets resulted in minimal differences, supporting the choice of the scenario using a 15% coverage target for ecoregions as an intermediate solution. The most divergent scenario in this category was derived from Jung et al.[6]. This scenario resulted in a smaller total affected population – approximately half compared to other scenarios in the group – and a higher proportion of the wild harvesting population. The distribution of populations by HDI, farm area, and livestock rangeland was broadly similar to other scenarios in the category. These results illustrate how variations in biodiversity representation targets, target-setting methods, and the inclusion or exclusion of specific features (e.g., ecoregions, KBAs) influence the spatial outcomes and, consequently, the social implications.

Results for the NCP approach scenarios presented greater variability than the other two scenario classes. Interestingly, the scenario based on Neugarten et al.'s[7] prioritisation integrating species representation and NCPs produced results that were closely aligned with the Biodiversity scenario. This scenario is a hybrid of the Biodiversity and NCP approaches, and this result reflects the shared input data between this scenario and the Biodiversity-based scenario, including species distributions. The scenario based on Chaplin-Kramer et al.'s[8] national-level prioritisation of NCPs closely matched the results of the primary layer presented in the main text despite different methodologies emphasising equitable distribution of contributions across countries. However, the most divergent results arose from Neugarten et al.'s[7] prioritisation run at a coarser 25 km^2 resolution. Despite using the same input data and methods as the main layer (4 km^2 resolution), this scenario resulted in a higher total affected population, reaching nearly 1.9 billion people in new areas and approximately 2.3 billion when including a 10 km buffer. Interestingly, the distribution of populations by HDI, wild harvesting, farm area, and livestock rangeland overlap in this lower-resolution variation of the scenario

remained relatively similar to other layers in the NCP category. This highlights the sensitivity of spatial prioritisation results to resolution differences, emphasising the critical role of data quality and methodological choices in influencing social outcomes.

For the ITT-based approach, the results from the alternative scenarios, both the one considering all mapped ITTs rather than a subsample (thus with a total coverage of 36%) and the one that focused solely on Indigenous peoples' data, were almost identical to those obtained with the primary layer presented in the main text. This scenario class consistently had the smallest resident and neighbouring populations, the highest proportion of people with low HDI, the smallest farm area, and the largest overlap with livestock rangeland areas.

These sensitivity analyses demonstrate the robustness of the primary scenario layers presented in the main text, indicating that our main results are consistent with variations in the construction of scenario layers while also highlighting how alternative datasets and methods can introduce meaningful variations.

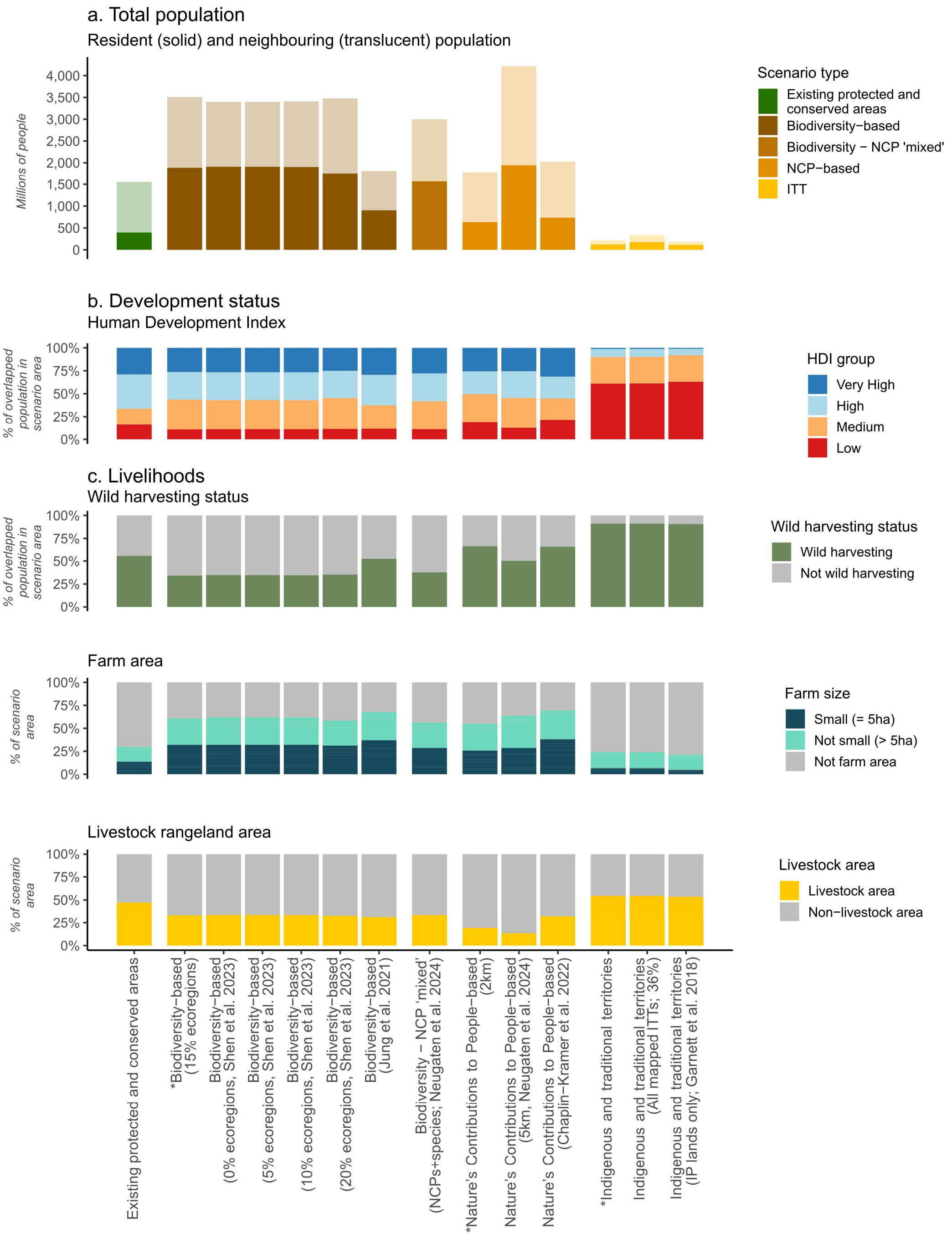

Supplementary Figure 8: Comparison of results for variation of scenarios in the Biodiversity-based, Nature's Contributions to People (NCP)-based and Indigenous and traditional territories scenario categories. Results shown for (a) total resident and neighbouring population in the scenario (Millions of people); (b) classification of population by Human Development Index group classes; and livelihoods, including wild harvest population (data for the tropics), farm area, classified by farm size, and livestock rangeland area. Scenarios tagged with an asterisk (*) in each category are those reported in the main text.

4.4 Sensitivity analysis of local wealth conditions

Although more diverse and higher resolution data are becoming available, mapping the wealth of population at scale remains challenging. Global and sub-global gridded poverty datasets often combine modelling and Earth Observation data to make high-resolution poverty predictions. Fine resolution is crucial for analyses such as ours or, for instance, the monitoring of Sustainable Development Goals (SDGs) and other trends in social well-being[9,10]. The training data used in these models typically originates from censuses, and in this process suffers from issues related to variable and limited spatio-temporal data coverage, incomparable data between countries, or low-quality data collection. Predictor variables often include satellite imagery, night-time lights, cell-phone connectivity, population data, and other socioeconomic and biophysical variables[11]. As a result, datasets often have limited accuracy, especially in regions with significant spatio-temporal livelihood and social system variations.

To assess these uncertainties, we complement results obtained for HDI[12], which provides a multidimensional perspective of development accounting for health, education, and income[13], with a comparison with three alternative poverty-related gridded datasets with sub-global coverage: the Relative Wealth Index[14] (RWI), the International Wealth Index[15] (IWI) and human well-being derived from unlit settlement footprint[16]. The countries included in the analysis for each metric are listed in Supplementary Table 6. For comparability, HDI-based results were recalculated for the same country subsets corresponding to each dataset.

We found that, in general, findings regarding the wealth of the population living in areas overlapped by our Target 3 scenarios hold true when considering these alternative metrics (Supplementary Fig. 9). Both RWI and the Wealth Index identify the ITT scenario as overlapping a larger proportion of less wealthy populations compared to current PAs and OECMs. For sub-Saharan Africa, HDI estimates show minimal discrimination among scenarios due to the large proportion of low HDI populations. However, IWI results align with the HDI findings while providing more detailed differentiation among scenarios. IWI values identify the ITT scenario as including a greater number of low-wealth populations and highlight the NCP-based scenario with a slightly larger overlap with populations in the lowest IWI percentile.

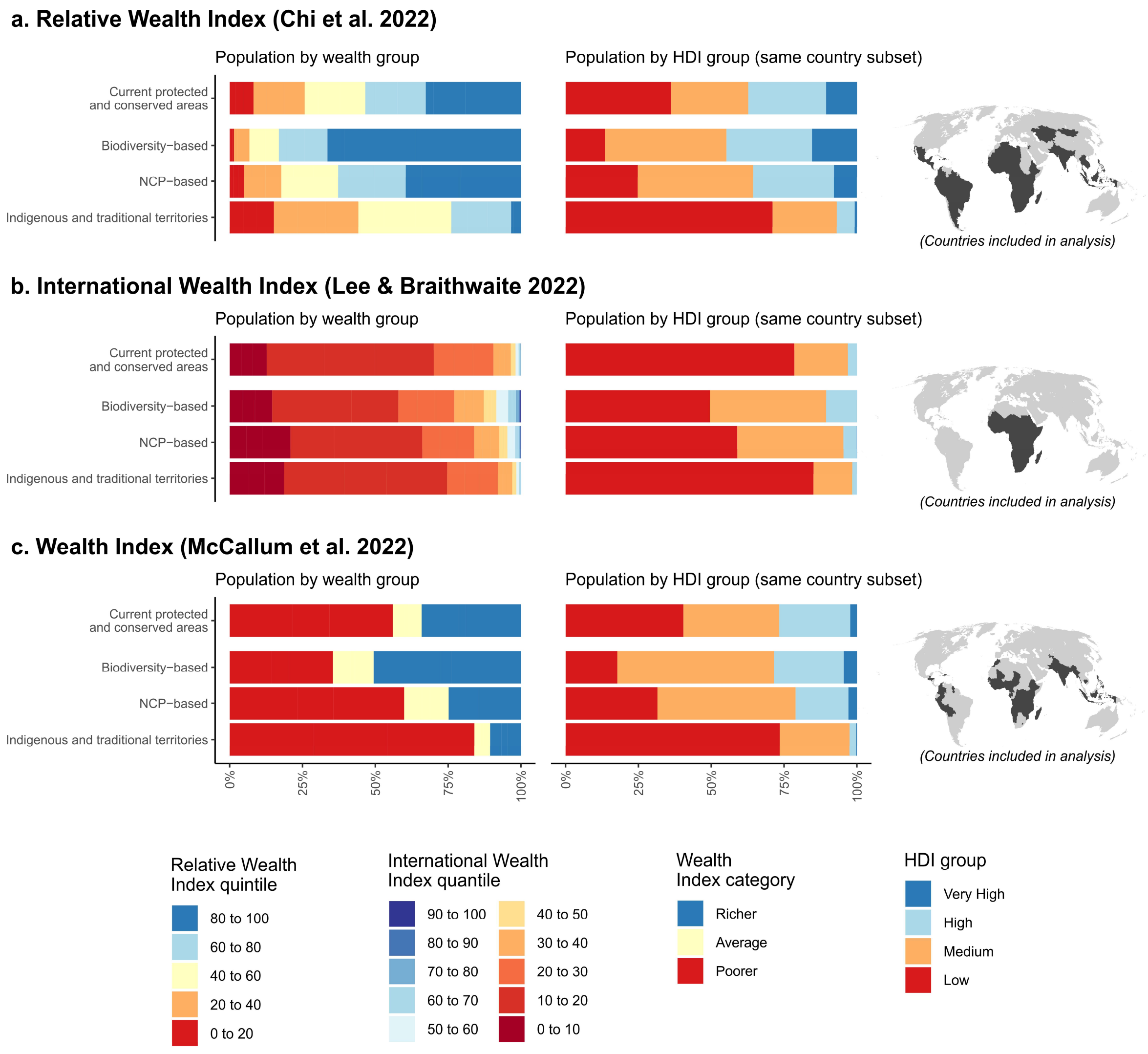

Supplementary Figure 9: Population in current protected and conserved areas and Target 3 scenarios according to three alternative wealth metrics: (a) Relative Wealth Index, (b) International Wealth Index and (c) Wealth Index by McCallum et al. (2022). For each wealth metric, the left bar plot shows the population by wealth group, while the right bar plot shows the corresponding population by Human Development Index (HDI) group recalculated for the same country subset. Maps in each row show the country coverage for each wealth metric: dark grey indicates countries included in the analysis (where data were available), and light grey indicates countries excluded. Both the wealth-based and HDI results within a row are calculated using only the countries shown in dark grey.

4.5 Sensitivity analysis of potential impact on livelihoods

To scrutinise variability in our main results with regard to livelihoods, we explored the dataset on land-use decision-making strategies by Malek & Verburg[17]. This dataset models the distribution of people by different land-use strategy classes. Among modelled classes, the group formed by survivalists, subsistence-oriented smallholders and market-oriented smallholders correspond to the classes that are more vulnerable to changes in land access. Other strategies (professional commercialists, professional intensifiers, eco-agriculturalists) were grouped. The data is provided at 10 km² resolution. We resampled the data to 25 km² and sampled it to match the Mollweide projection of our scenarios for analysis.

The ITT scenario shows the highest proportion of people from the survivalist and smallholder strategies amongst those overlapped. More than 75% of the overlapped population are estimated to rely on these strategies, especially survivalists and subsistence-oriented smallholders (Supplementary Fig. 10). The NCP scenario overlaps with 45%-50% of such populations, at the same level of current protected and conserved areas. In contrast, the Biodiversity-based scenario is the one with the smallest proportional overlap with populations using survivalist or any of the two smallholder strategies.

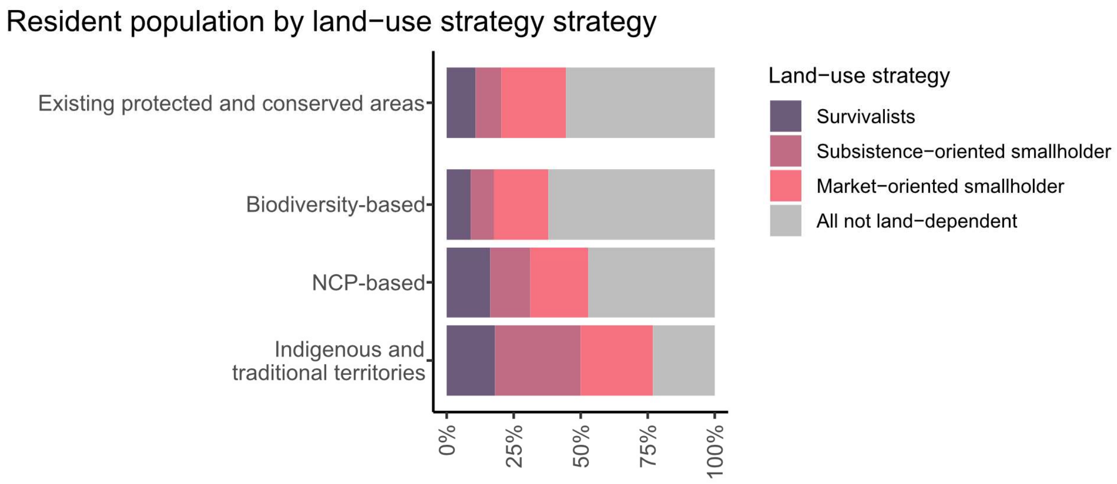

Supplementary Figure 10: Resident population in existing protected and conserved areas and Target 3 scenarios by their most likely land-use strategy.

4.6 Sensitivity analysis of scenario neighbouring areas

The positive and negative influence of area-based conservation measures extend beyond those living directly within current protected and conserved areas, particularly affecting those living in close proximity to them. Ten km represents the area where most socioeconomic impacts of area-based conservation, both positive and negative, are locally felt[18,19]. However, it is expected that this distance is different across types of impacts and geographies. To account for this, we estimated the population living within multiple distances of area-based conservation sites in the scenarios.

According to our datasets (GHS-POP[1], estimates for the year 2023), the population within 15 km of current protected and conserved areas, including those residing within these areas, amounts to 2.3 billion people, approximately 30.7% of the global population (Supplementary Fig. 11). This percentage decreases to 20.3% (1.6 billion people) and 11.9% (913 million people) when considering only a 10 km or 5 km buffer, respectively. From evaluated scenarios, maximising biodiversity representation encompasses the largest resident and neighboring population, with new areas under this scenario covering 50% of the global population within a 15 km buffer (including expanded areas). When aggregated with the population within existing protected and conserved areas and their 15 km buffer, this results in a map involving over 80% of the global population. At the opposite end of the spectrum is the Indigenous and traditional territories scenario, which includes 368 million people (just over 4.8% of the global population) within expanded areas and a 15 km buffer – slightly fewer than the number currently residing within boundaries of existing protected and conserved areas. The scenario focused on maximising NCPs falls in between, encompassing a total population of 2 billion people within a 15 km buffer, slightly less than the population within the same distance of existing protected and conserved areas.

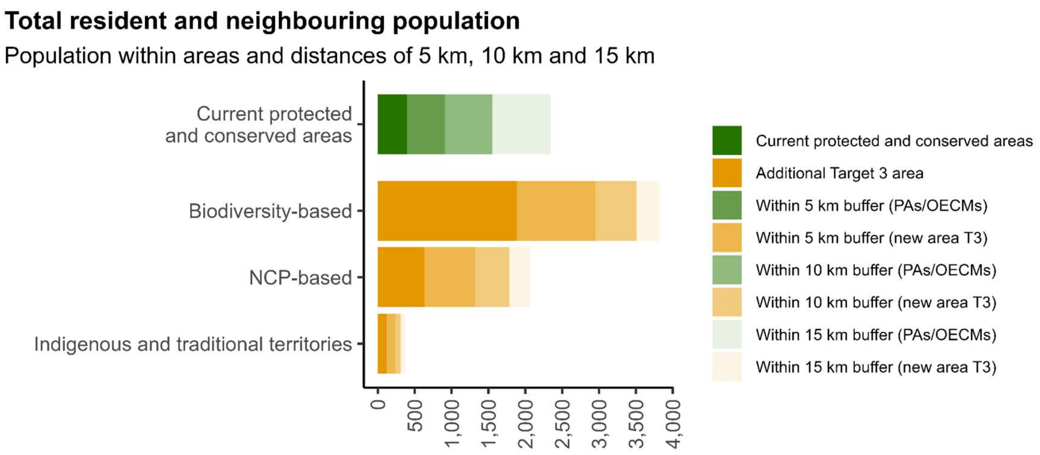

Supplementary Figure 11: Population residing within current protected and conserved areas (green), new Target 3 (T3) scenario areas (orange), and within 5 km, 10 km and 15 km buffers of these areas.

Supplementary Tables

Supplementary Table 1: Population in current protected and conserved areas and Target 3 scenarios.

Supplementary Table 2: Population in scenarios by continent.

Supplementary Table 3: Population in current protected and conserved areas and Target 3 scenarios classified by Human Development Index.

Supplementary Table 4: Livelihood overlap with current protected and conserved areas and Target 3 scenarios.

Supplementary Table 5: Datasets and underlying input data used in the protected area and OECM baseline dataset, Target 3 scenarios and the spatial analyses of local social conditions.

Supplementary Table 1

Population in scenarios expressed as totals and as a percentage of the global population by 2023.

**Scenario**	Population in scenario (millions)	% of global population	Population in existing protected and conserved areas and scenario (millions)	% of global population
Existing protected and conserved areas				
Within<br>*Buffer area*<br>*Combined*	396<br>1157<br>1554	5.2 %<br>15.2 %<br>20.3 %		
Biodiversity-based scenario				
Within<br>*Buffer area*<br>*Combined*	1887<br>1624<br>3511	24.7 %<br>21.3 %<br>46.0 %	2283<br>2782<br>5065	29.9 %<br>36.4 %<br>66.3 %
NCP-based scenario				
Within<br>*Buffer area*<br>*Combined*	633<br>1148<br>1782	8.3 %<br>15.0 %<br>23.3 %	1030<br>2310<br>3803	13.5 %<br>30.2 %<br>49.8 %
Indigenous and Traditional Territories scenario				
Within<br>*Buffer area*<br>*Combined*	120 ± 0.7<br>188 ± 0.9<br>309 ± 1.6	1.6 %<br>2.5 %<br>4.0 %	517 ± 0.7<br>1346 ± 0.9<br>1853 ± 1.6	6.8 %<br>17.6 %<br>24.3%

Supplementary Table 2

Population in Target 3 scenarios by continent.

**Scenario**	Population in scenario (millions)
Africa	
Existing protected and conserved areas	90.0
Biodiversity-based scenario	383.4
NCP-based scenario	165.9
Indigenous and Traditional Territories scenario	76.1
Asia	
Existing protected and conserved areas	174.4
Biodiversity-based scenario	1029.0
NCP-based scenario	237.5
Indigenous and Traditional Territories scenario	35.5
Australia and Oceania	
Existing protected and conserved areas	0.8
Biodiversity-based scenario	20.6
NCP-based scenario	6.2
Indigenous and Traditional Territories scenario	0.05
Europe	
Existing protected and conserved areas	64.7
Biodiversity-based scenario	74.3
NCP-based scenario	80.3
Indigenous and Traditional Territories scenario	0.1
North and Central America	
Existing protected and conserved areas	16.3
Biodiversity-based scenario	175.3
NCP-based scenario	72.4
Indigenous and Traditional Territories scenario	5.0
South America	
Existing protected and conserved areas	50.1
Biodiversity-based scenario	201.0
NCP-based scenario	70.7
Indigenous and Traditional Territories scenario	3.6

Supplementary Table 3

Population in scenarios classified by Human Development Index group, and expressed as totals and as a percentage of the global population by 2023. Low HDI: <0.550; Medium HDI: 0.550-0.699; High HDI: 0.700-0.799; Very High HDI: >0.800.

**Scenario**		Population in scenario (millions)	% of global population in HDI group
Existing protected and conserved areas			
Within	*Low HDI areas*	61.8	6.8 %
	Medium HDI areas	67.4	2.9 %
	High HDI areas	142.5	6.4 %
	Very high HDI areas	110.6	5.7 %
Buffer	*Low HDI areas*	122.6	13.4 %
	Medium HDI areas	230.3	10.0 %
	High HDI areas	303.5	13.6 %
	Very high HDI areas	467.1	24.2 %
Biodiversity-based scenario			
Within	*Low HDI areas*	195.2	21.4 %
	Medium HDI areas	596.3	26.0 %
	High HDI areas	545.0	24.3 %
	Very high HDI areas	476.5	24.7 %
Buffer	*Low HDI areas*	166.4	18.2 %
	Medium HDI areas	518.5	22.6 %
	High HDI areas	493.6	22.0 %
	Very high HDI areas	381.1	19.8 %
NCP-based scenario			
Within	*Low HDI areas*	118.0	12.9 %
	Medium HDI areas	188.3	8.2 %
	High HDI areas	152.9	6.8 %
	Very high HDI areas	158.3	24.7 %
Buffer	*Low HDI areas*	147.8	16.2 %
	Medium HDI areas	335.2	14.6 %
	High HDI areas	303.7	13.6 %
	Very high HDI areas	324.0	16.8 %
Indigenous and Traditional Territories scenario			
Within	*Low HDI areas*	73.7 ± 0.6	3.2 %
	Medium HDI areas	34.8 ± 0.3	1.5 %
	High HDI areas	10.3 ± 0.1	1.1 %
	Very high HDI areas	1.0 ± 0.0	0.1 %
Buffer	*Low HDI areas*	86.7 ± 0.7	3.8 %
	Medium HDI areas	58.2 ± 0.4	2.6 %
	High HDI areas	32.9 ± 0.4	3.6 %
	Very high HDI areas	8.5 ± 0.2	0.4 %

Supplementary Table 4

Livelihood overlap with current protected and conserved areas and Target 3 scenarios. Results are provided for the three evaluated livelihoods: wild harvesting, farm area, and livestock keeping. For each livelihood, we report the population or area potentially affected within the new areas, neighboring areas (within 10 km), and combined area, and as proportion of the population or total area overlapped. HH: households.

**Wild Harvesting**				
**Scenario**	Population from wild harvesting HH in scenario (millions)	% of scenario population	Population from HH not wild harvesting in scenario (millions)	% of scenario population
Existing protected and conserved areas				
Within	111.9	55.6 %	89.3	44.4 %
Buffer area	211.0	46.9 %	238.8	53.1 %
Combined	323.0	49.6 %	328.1	50.4 %
Biodiversity-based scenario				
Within	336.9	34.1 %	649.7	65.8 %
Buffer area	290.3	39.8 %	438.9	60.2 %
Combined	627.2	36.5 %	1088.6	63.4 %
NCP-based scenario				
Within	275.4	66.7 %	137.6	33.3 %
Buffer area	272.8	48.4 %	290.9	51.6 %
Combined	548.2	56.1 %	428.5	43.9 %
Indigenous and traditional territories scenario				
Within	82.2 ± 0.3	91.2 %	7.9 ± 0.0	8.8 %
Buffer area	20.6 ± 0.2	17.3 %	98.2 ± 0.4	82.7 %
Combined	102.8 ± 0.5	49.2 %	106.1 ± 0.4	50.9 %

**Farm area**				
**Scenario**	Farm area (millions of km^2)	% of all scenario area	Small farm area (≤5ha) (millions of km2)	% of all scenario area
Existing protected and conserved areas				
Within	6.9	29.8 %	3.7	16.2 %
Buffer area	8.4	54.9 %	3.8	25.0 %
Combined	15.3	39.7 %	7.6	19.7 %

Biodiversity-based scenario				
Within<br>*Buffer area*<br>*Combined*	10.3<br>10.2<br>20.6	60.8 %<br>58.9 %<br>59.8 %	4.9<br>4.6<br>9.5	28.8 %<br>26.3 %<br>27.5 %
NCP-based scenario				
Within<br>*Buffer area*<br>*Combined*	9.4<br>7.6<br>16.9	54.9 %<br>67.1 %<br>59.8 %	5.0<br>3.5<br>8.5	29.2 %<br>31.3 %<br>30.0 %
Indigenous and traditional territories scenario				
Within<br>*Buffer area*<br>*Combined*	1.1 ± 1.1<br>0.9 ± 0.9<br>2.0 ± 1.9	8.0 %<br>10.4 %<br>8.9 %	1.0 ± 0.9<br>0.8 ± 0.7<br>1.7 ± 1.6	7.0 %<br>8.6 %<br>7.6 %

**Livestock rangeland area**		
**Scenario**	Livestock area (millions of km^2)	% of all scenario area
Existing protected and conserved areas		
Within<br>*Buffer area*<br>*Combined*	10.8<br>4.9<br>15.8	47.3 %<br>32.1 %<br>41.3 %
Biodiversity-based scenario		
Within<br>*Buffer area*<br>*Combined*	5.7<br>6.4<br>12.0	33.4 %<br>37.0 %<br>35.2 %
NCP-based scenario		
Within<br>*Buffer area*<br>*Combined*	3.3<br>2.4<br>5.7	19.3 %<br>21.3 %<br>20.1 %
Indigenous and traditional territories scenario		
Within<br>*Buffer area*<br>*Combined*	9.1 ± 0.0<br>5.9 ± 0.0<br>15.0 ± 0.0	54.4 %<br>51.5 %<br>53.2 %

Supplementary Table 5

Datasets and underlying input data used in the protected area and OECM baseline dataset, Target 3 scenarios and the spatial analyses of local social conditions. The table includes, for each dataset, their sources and description. Indentation denotes underlying data.

Dataset	Source	Description
**Existing protected and conserved areas**		
WDPA and WD-OECM	20	Current protected and conserved areas. Dataset for August 2024. Protected areas and OECMs from China extracted from Shen et al.[5] for March 2020. Protected areas and OECMs from India obtained from Protected Planet from September 2019 (last month areas were publicly available)
**Biodiversity-based scenario**		
Global Terrestrial Ecoregions	21	Used as feature to represent in spatial prioritisation
Key Biodiversity Areas	22	Unprotected KBAs included in solutions of spatial prioritisation
Species' Area of Habitat (AOH) maps		Includes 5,520 mammals, 10,936 birds, 6,594 amphibians and 7,042 reptiles. Used as features to represent in spatial prioritisation
Geographic range maps for species distributions	23	Basis of species' AOHs.
Habitats	24	Used to produce AOHs to remove areas in unsuitable habitat types for each species. Resolution: ~3.5 arc-sec (~110 m)
Elevation	25	Used to produce AOHs to remove areas in unsuitable elevations for each species. Resolution: 3 arc-sec (~90 m)
**NCP-based scenario**		
Scenario raster	7	
Nitrogen retention for water quality regulation (NCP)	8	Used as feature to represent in spatial prioritisation Modelled using InVEST. Original resolution: 10 arc-sec (~300 m)
Sediment retention for water quality regulation (NCP)	8	Used as feature to represent in spatial prioritisation Modelled using InVEST. Original resolution: 10 arc-sec (~300 m)
Crop pollination contribution to nutrition production (NCP)	8	Used as feature to represent in spatial prioritisation Modelled using InVEST. Original resolution: 10 arc-sec (~300 m)

Fodder production for livestock (NCP)	8,26	Used as feature to represent in spatial prioritisation<br>Modelled using Co$ting Nature. Original resolution: 5 arc-min (~10 km)
Timber production (commercial and domestic) (NCP)	8,26	Used as feature to represent in spatial prioritisation<br>Modelled using Co$ting Nature. Original resolution: 5 arc-min (~10 km)
Fuel wood production (NCP)	8,26	Used as feature to represent in spatial prioritisation<br>Modelled using Co$ting Nature. Original resolution: 5 arc-min (~10 km)
Flood regulation (NCP)	8,27,28	Used as feature to represent in spatial prioritisation<br>Modelled with WaterWorld. Original resolution: 5 arc-min (~10 km)
Access to nature (habitat within one hour of rural and urban populations) (NCP)	8	Used as feature to represent in spatial prioritisation.<br>Original resolution: 10 arc-sec (~300 m)
Vulnerable ecosystem carbon storage (NCP)	29	Used as feature to represent in spatial prioritisation.<br>Original resolution: 1 arc-sec (~30 m)
Coastal risk reduction (NCP)	8	Used as feature to represent in spatial prioritisation.<br>Modelled using InVEST. Original resolution: 10 arc-sec (~300 m)
Land cover (ESA Climate Change Initiative)	30	Used to mask NCP layers to exclude cropland, mosaic cropland, urban areas, bare areas, water bodies, permanent snow & ice.<br>Original resolution: 10 arc-sec (~300 m)
**Indigenous and traditional territories**		
Indigenous and Community Conserved Areas (ICCAs)	31	ICCAs are areas where Indigenous peoples and local communities protect and care for lands, waters and collective territories. They are included in the scenario area.
Indigenous Peoples (IPs) and Local Communities (LCs) lands	32	Sites included in the scenario area.
Australia's Indigenous land	33	Sites included in the scenario area, replacing IP and LC lands from [32] in Australia.
Human modification Index (year 2017)	34	Used to focus scenario on areas with high biodiversity value. HMI measures the intensity and spatial extent of anthropogenic pressures on landscapes, integrating factors such as urbanization, agriculture, infrastructure development and other land uses. Used to eliminate areas with a human modification above 0.1.

**Social datasets**		
Global Human Settlement Population (GHS-POP) (year 2023)	1	
Sentinel/Landsat based GHS-BUILT-V		Used as target for disaggregation of population estimates.
Gridded Population of the World, version 4.11 (GPWv4.11)		Used as base source for population estimates (both census unit counts and geometries)
Gridded municipality-level Human Development Index	12	
Daytime imagery features created from Planet's Surface Reflectance Basemaps, year 2019	35	Used as features in machine learning modelling frameworks
Nightlights features created from Defense Meteorological Satellite Program (DMSP), year 2013	36	Used as features in machine learning modelling frameworks
Wild Harvesting	37	
Household's survey data	38–41	Used in modelling as response (self-reported harvests during the period) and contextual variables (cultivation presence, other income presence, productive asset presence, education, male household head, wealth rank, food security, life satisfaction, regulated common-pool resources presence.<br>Data from 10,793 households, representative of 438 villages in 24 low- and middle-income countries, spanning Latin America, sub-Saharan Africa, South Asia, and South East Asia.
% natural LC within 5km	42	Use in modelling as contextual variable
Distance to nearest city	2	Use in modelling as contextual variable
Stable night light intensity	43	Use in modelling as contextual variable
WorldPop's Human population count estimate	2	Used to generate estimates of number of people wild harvesting; 1 km resolution global gridded population count estimates for the year 2015 (unconstrained top-down global mosaics suitable for areas with many small rural settlements).
Farm area classified by field size	44	
Field size	45	Crowd-sourced field size estimates, used to inform field size

Cropland area	46	Spatially explicit dataset on cropland area, used by Mehrabi et al.[44] to train the farm size model.
**Livestock rangeland area**	47	
Land cover (GLC 2000)	48	Used in the building of the livestock land system layer to define areas of pasture, rangelands within them, cropland, and mixed areas.
Human population density year 2000	49	Used in the building of the livestock land system layer to identify cropping areas within the cultivatable rangelands category, which are excluded in the livestock rangeland system subtype.
Length of growing period (LGP)	50	Used in the building of the livestock land system layer to classify rangelands into "cultivatable" and "non-cultivatable".
Irrigated areas	51	Based on Aquastat Version 4.0.1 (source data at 1 km), this data is used in the building of the livestock land system layer to identify irrigated rangelands (excluded from the livestock rangeland area category)

Supplementary Table 6

List of countries for which data were available and included in the sensitivity analysis of local wealth conditions, using the Relative Wealth Index (RWI), International Wealth Index (IWI), and unlit settlement footprint well-being datasets.

**Metric**	**Countries and territories included in analysis***
Relative Wealth Index (RWI)	Algeria, Angola, Argentina, Bangladesh, Belize, Benin, Bhutan, Bolivia (Plurinational State of), Botswana, Brazil, Burkina Faso, Burundi, Cabo Verde, Cambodia, Cameroon, Central African Republic, Chad, Colombia, Comoros, Congo, Costa Rica, Côte d'Ivoire, Democratic Republic of the Congo, Djibouti, Dominica, Dominican Republic, Ecuador, El Salvador, Equatorial Guinea, Eritrea, Eswatini, Ethiopia, Gabon, Gambia, Ghana, Grenada, Guatemala, Guinea, Guinea-Bissau, Guyana, Haiti, Honduras, India, Indonesia, Jamaica, Jordan, Kazakhstan, Kenya, Kyrgyzstan, Lao People's Democratic Republic, Lesotho, Liberia, Libya, Madagascar, Malawi, Malaysia, Maldives, Mali, Mauritania, Mauritius, Mexico, Mongolia, Mozambique, Namibia, Nepal, Nicaragua, Niger, Nigeria, Paraguay, Peru, Philippines, Rwanda, Saint Lucia, Saint Vincent and the Grenadines, Sao Tome and Principe, Senegal, Sierra Leone, South Africa, Sri Lanka, Suriname, Tajikistan, Thailand, Timor-Leste, Togo, Tunisia, Turkmenistan, Uganda, United Republic of Tanzania, Uzbekistan, Viet Nam, Zambia, Zimbabwe.
International Wealth Index (IWI)	Angola, Benin, Botswana, Burkina Faso, Burundi, Cameroon, Central African Republic, Chad, Congo, Côte d'Ivoire, Democratic Republic of the Congo, Djibouti, Equatorial Guinea, Eritrea, Eswatini, Ethiopia, Gabon, Gambia, Ghana, Guinea, Guinea-Bissau, Kenya, Lesotho, Liberia, Madagascar, Malawi, Mali, Mauritania, Mozambique, Namibia, Niger, Nigeria, Rwanda, Senegal, Sierra Leone, Somalia, South Africa, South Sudan, Sudan, Togo, Uganda, United Republic of Tanzania, Zambia, Zimbabwe.
Unlit settlement footprint well-being	Angola, Bangladesh, Benin, Bolivia (Plurinational State of), Burkina Faso, Burundi, Cambodia, Cameroon, Chad, Colombia, Comoros, Côte d'Ivoire, Democratic Republic of the Congo, Dominican Republic, Ethiopia, Gabon, Ghana, Guatemala, Guinea, Guyana, Haiti, Honduras, India, Indonesia, Kenya, Lesotho, Liberia, Madagascar, Malawi, Mali, Morocco, Mozambique, Myanmar, Namibia, Nepal, Nigeria, Pakistan, Peru, Philippines, Rwanda, Senegal, Sierra Leone, Tajikistan, Timor-Leste, Togo, Uganda, United Republic of Tanzania, Zambia, Zimbabwe.

**The names and designation used on this table do not imply official endorsement or acceptance by the United Nations.*

Supplementary References

1. European Commission & Joint Research Centre. *GHSL Data Package 2023.* (Publications Office, LU, 2023).

2. Lloyd, C. T. *et al.* Global spatio-temporally harmonised datasets for producing high-resolution gridded population distribution datasets. *Big Earth Data* **3**, 108–139 (2019).

3. CIESIN & Columbia University. Documentation for the Gridded Population of the World, Version 4 (GPWv4), Revision 11 Data Sets. https://doi.org/10.7927/H45Q4T5F (2018) doi:10.7927/H45Q4T5F.

4. Dobson, J. E., Bright, E. A., Coleman, P. R., Durfee, R. C. & Worley, B. A. LandScan: A global population database for estimating populations at risk. *Photogrammetric Engineering and Remote Sensing* **66**, 849–857 (2000).

5. Shen, X. *et al.* Countries' differentiated responsibilities to fulfill area-based conservation targets of the Kunming-Montreal Global Biodiversity Framework. *One Earth* **6**, 548–559 (2023).

6. Jung, M. *et al.* Areas of global importance for conserving terrestrial biodiversity, carbon and water. *Nat Ecol Evol* **5**, 1499-1509 (2021).

7. Neugarten, R. A. *et al.* Mapping the planet's critical areas for biodiversity and nature's contributions to people. *Nat Commun* **15**, 261 (2024).

8. Chaplin-Kramer, R. *et al.* Mapping the planet's critical natural assets. *Nat Ecol Evol* **7**, 51–61 (2023)

9. IEAG. *A World That Counts: Mobilising the Data Revolution for Sustainable Development. Independent Expert Advisory Group on a Data Revolution for Sustainable Development*. https://www.undatarevolution.org/wp-content/uploads/2014/11/A-World-That-Counts.pdf (2014).

10. Watmough, G. R. *et al.* Socioecologically informed use of remote sensing data to predict rural household poverty. *Proceedings of the National Academy of Sciences* **116**, 1213–1218 (2019).

11. Jean, N. *et al.* Combining satellite imagery and machine learning to predict poverty. *Science* **353**, 790–794 (2016).

12. Sherman, L., Proctor, J., Druckenmiller, H., Tapia, H. & Hsiang, S. M. Global High-Resolution Estimates of the United Nations Human Development Index Using Satellite Imagery and Machine-learning. Working Paper at https://doi.org/10.3386/w31044 (2023).

13. Kovacevic, M. *Review of HDI Critiques and Potential Improvements*. *Human Development Reports* https://hdr.undp.org/content/review-hdi-critiques-and-potential-improvements (2010).

14. Chi, G., Fang, H., Chatterjee, S. & Blumenstock, J. E. Microestimates of wealth for all low- and middle-income countries. *Proceedings of the National Academy of Sciences* **119**, e2113658119 (2022).

15. Lee, K. & Braithwaite, J. High-resolution poverty maps in Sub-Saharan Africa.

World Development **159**, 106028 (2022).

16. McCallum, I. *et al.* Estimating global economic well-being with unlit settlements. *Nat Commun* **13**, 2459 (2022).
17. Malek, Ž. & Verburg, P. H. Mapping global patterns of land use decision-making. *Global Environmental Change* **65**, 102170 (2020).
18. Naidoo, R. *et al.* Evaluating the impacts of protected areas on human well-being across the developing world. *Science Advances* **5**, eaav3006 (2019).
19. Oldekop, J. A., Holmes, G., Harris, W. E. & Evans, K. L. A global assessment of the social and conservation outcomes of protected areas. *Conservation Biology* **30**, 133–141 (2016).
20. UNEP-WCMC & IUCN. *Protected Planet: The World Database on Protected Areas (WDPA) and Database on Other Effective Area-Based Conservation Measures (WD-OECM), August 2024*. (UNEP-WCMC and IUCN, UK, 2024).
21. Dinerstein, E. *et al.* An Ecoregion-Based Approach to Protecting Half the Terrestrial Realm. *BioScience* **67**, 534–545 (2017).
22. IUCN. A global standard for the identification of Key Biodiversity Area. IUCN (2016).
23. IUCN. *Red List of Threatened Species: Version 2019.9*. https://www.iucnredlist.org/ (2019).
24. Lumbierres, M. *et al.* Translating habitat class to land cover to map area of habitat of terrestrial vertebrates. *Conservation Biology* **36**, (2022).
25. Robinson, N., Regetz, J. & Guralnick, R. P. EarthEnv-DEM90: A nearly-global, void-free, multi-scale smoothed, 90m digital elevation model from fused ASTER and SRTM data. *ISPRS Journal of Photogrammetry and Remote Sensing* **87**, 57–67 (2014).
26. Mulligan, M. Documentation for the Co$tingNature Model V3. www.policysupport.org/costingnature. Original V3 documentation 2017. (2018).
27. Gunnell, K., Mulligan, M., Francis, R. A. & Hole, D. G. Evaluating natural infrastructure for flood management within the watersheds of selected global cities. *Science of The Total Environment* **670**, 411–424 (2019).
28. Mulligan, M. Documentation for the WaterWorld Model V2. www.policysupport.org/waterworld; https://docs.google.com/document/d/1GKheQFp5_rsZyazwCJxCzeEStQF2jh04x-Dl_oG5yoY/mobilebasic (2022).
29. Noon, M. L. *et al.* Mapping the irrecoverable carbon in Earth's ecosystems. *Nat Sustain* **5**, 37–46 (2021).
30. Defourny, P. *et al. Observed Annual Global Land-Use Change from 1992 to 2020 Three Times More Dynamic than Reported by Inventory-Based Statistics.* (2017).
31. UNEP-WCMC. ICCA Registry [On-line], November 2023, Cambridge, UK: UNEP-WCMC. (2023).

32. WWF *et al. The State of Indigenous Peoples' and Local Communities' Lands and Territories: A Technical Review of the State of Indigenous Peoples' and Local Communities' Lands, Their Contributions to Global Biodiversity Conservation and Ecosystem Services, the Pressures They Face, and Recommendations for Actions*. https://wwflac.awsassets.panda.org/downloads/report_the_state_of_the_indigenous_peoples_and_local_communities_lands_and_territories_1.pdf (2021).

33. Jacobsen, R., Howell, C. & Read, S. *Australia's Indigenous land and forest estate: separate reporting of Indigenous ownership, management and other special rights* (ABARES, 2020) https://doi.org/10.25814/BQR0-4M20

34. Theobald, D. M. *et al.* Earth transformed: detailed mapping of global human modification from 1990 to 2017. *Earth System Science Data* **12**, 1953–1972 (2020).

35. Planet. Planet Application Program Interface: In Space for Life on Earth. https://api.planet.com (2017).

36. Payne Institute for Public Policy. DMSP-OLS Nighttime Lights Time Series (Version 4). https://eogdata.mines.edu/products/dmsp/%5C#v4%5C_dmsp%5C_download (2021).

37. Wells, G. J. *et al.* Hundreds of millions of people in the tropics need both wild harvests and other forms of economic development for their well-being. *One Earth* **7**, 311–324 (2024).

38. CIFOR. CIFOR's Poverty and Environment Network (PEN) Global Dataset, V2. Center for International Forestry Research (CIFOR) (2016).

39. Devagiri, G. M. *et al.* Western Ghats Household Baseline. *ATREE* https://agris.fao.org/search/en/providers/123417/records/6474613e425ec3c088ede31c (2015).

40. Robinson, B. E., Zheng, H. & Peng, W. Disaggregating livelihood dependence on ecosystem services to inform land management. *Ecosystem Services* **36**, 100902 (2019).

41. Schreckenberg, K., Mace, G. & Poudyal, M. *Ecosystem Services and Poverty Alleviation: Trade-Offs and Governance*. (2018).

42. ESA. Land Cover CCI Product Version 2. Climate Change Initiative, Paris (2017).

43. Li, X., Zhou, Y., Zhao, M. & Zhao, X. A harmonized global nighttime light dataset 1992–2018. *Sci Data* **7**, 168 (2020).

44. Mehrabi, Z. *et al.* The global divide in data-driven farming. *Nat Sustain* **4**, 154–160 (2021).

45. Lesiv, M. *et al.* Estimating the global distribution of field size using crowdsourcing. *Global Change Biology* **25**, 174–186 (2019).

46. Herrero, M. *et al.* Farming and the geography of nutrient production for human use: a transdisciplinary analysis. *The Lancet Planetary Health* **1**, e33–e42 (2017).

47. Robinson, T. P. *et al.* Global distribution of ruminant livestock production systems V5 (5 minutes of arc). Harvard Dataverse https://doi.org/10.7910/DVN/WPDSZE (2018).

48. Bartholomé, E. & Belward, A. S. GLC2000: a new approach to global land cover mapping from Earth observation data. *International Journal of Remote Sensing* **26**, 1959–1977 (2005).

49. CIESIN. *Global Rural–Urban Mapping Project (GRUMP)*. (2005).

50. Thornton, P. K. & Jones, P. G. *Global Length of Growing Period for Current Conditions at 1km Resolution*. (2010).

51. Siebert, S., Döll, P., Feick, S., Hoogeveen, J. & Frenken, K. *Global Map of Irrigated Areas Version 4.0.1.* (2007).
