# Supplementary material for: Social implications of the 30×30 global conservation target
Source: Nat Commun. 2026 May 12;17:4067. doi: 10.1038/s41467-026-71860-8 (PMC13168258; doi:10.1038/s41467-026-71860-8)
Supplement: Supplementary file 2 — Reporting Summary [file 41467_2026_71860_MOESM2_ESM.pdf]

nature portfolio

Corresponding author(s): Javier Fajardo and Chris Sandbrook

Last updated by author(s): Nov 21, 2025

Reporting Summary

Nature Portfolio wishes to improve the reproducibility of the work that we publish. This form provides structure for consistency and transparency in reporting. For further information on Nature Portfolio policies, see our Editorial Policies and the Editorial Policy Checklist.

Statistics

For all statistical analyses, confirm that the following items are present in the figure legend, table legend, main text, or Methods section.

n/a	Confirmed	
☐	☒	The exact sample size (*n*) for each experimental group/condition, given as a discrete number and unit of measurement
☒	☐	A statement on whether measurements were taken from distinct samples or whether the same sample was measured repeatedly
☒	☐	The statistical test(s) used AND whether they are one- or two-sided<br>*Only common tests should be described solely by name; describe more complex techniques in the Methods section.*
☒	☐	A description of all covariates tested
☒	☐	A description of any assumptions or corrections, such as tests of normality and adjustment for multiple comparisons
☐	☒	A full description of the statistical parameters including central tendency (e.g. means) or other basic estimates (e.g. regression coefficient) AND variation (e.g. standard deviation) or associated estimates of uncertainty (e.g. confidence intervals)
☒	☐	For null hypothesis testing, the test statistic (e.g. *F*, *t*, *r*) with confidence intervals, effect sizes, degrees of freedom and *P* value noted<br>*Give P values as exact values whenever suitable.*
☐	☐	For Bayesian analysis, information on the choice of priors and Markov chain Monte Carlo settings
☒	☐	For hierarchical and complex designs, identification of the appropriate level for tests and full reporting of outcomes
☒	☐	Estimates of effect sizes (e.g. Cohen's *d*, Pearson's *r*), indicating how they were calculated

Our web collection on statistics for biologists contains articles on many of the points above.

Software and code

Policy information about availability of computer code

Data collection	No software was used for data collection
Data analysis	The analyses were conducted using R (version 4.4.2), with key packages including prioritizr (v8.1.0), terra (v1.8-54), tidyverse (v2.0.0) and tidyterra (v0.7.2).<br>The R scripts used for data processing, analysis, and figure generation in this study are publicly available at https://doi.org/10.5281/zenodo.18344001, under GPL-3 license.

For manuscripts utilizing custom algorithms or software that are central to the research but not yet described in published literature, software must be made available to editors and reviewers. We strongly encourage code deposition in a community repository (e.g. GitHub). See the Nature Portfolio guidelines for submitting code & software for further information.

Data

Policy information about availability of data

All manuscripts must include a data availability statement. This statement should provide the following information, where applicable:

- Accession codes, unique identifiers, or web links for publicly available datasets
- A description of any restrictions on data availability
- For clinical datasets or third party data, please ensure that the statement adheres to our policy

This study did not generate or collect any new data. All data used in the analyses were obtained from publicly available sources and are cited in the manuscript. Readers can access the original datasets through the referenced sources.

Research involving human participants, their data, or biological material

Policy information about studies with human participants or human data. See also policy information about sex, gender (identity/presentation), and sexual orientation and race, ethnicity and racism.

Reporting on sex and gender	This information has not been collected
Reporting on race, ethnicity, or other socially relevant groupings	No race or ethnicity variables were used in this study. All social variables were derived from publicly available, aggregate global datasets (e.g., UNDP Human Development Index, WorldPop, and global livelihood indicators). These datasets were produced by third parties using their own documented methodologies and are available in the public domain, with access information provided in our manuscript, and no reclassification of individuals or social groups was undertaken by the authors. The variables were included to characterise broad socioeconomic conditions relevant to potential conservation expansion and were not used as proxies for race, ethnicity, or other sensitive identities. As the analysis is descriptive and spatial rather than causal, controlling for confounding variables was not applicable.
Population characteristics	This study did not involve human research participants. All analyses were based on aggregate, non-identifiable socioeconomic and demographic datasets produced by third-party providers. Therefore, no individual-level population characteristics apply.
Recruitment	This study did not involve human research participants. All analyses used publicly available, aggregate datasets from third-party sources, so no recruitment procedures were applicable.
Ethics oversight	This study did not involve human research participants, interventions, or identifiable personal data; therefore, no institutional ethics approval of protocols was required.

Note that full information on the approval of the study protocol must also be provided in the manuscript.

Field-specific reporting

Please select the one below that is the best fit for your research. If you are not sure, read the appropriate sections before making your selection.

☐ Life sciences ☐ Behavioural & social sciences ☒ Ecological, evolutionary & environmental sciences

For a reference copy of the document with all sections, see nature.com/documents/nr-reporting-summary-flat.pdf

Ecological, evolutionary & environmental sciences study design

All studies must disclose on these points even when the disclosure is negative.

Study description	This study provides a global, spatially explicit assessment of the social conditions in areas that may be designated as protected or conserved under three alternative site-selection scenarios for achieving Target 3 of the Kunming–Montreal Global Biodiversity Framework. The analysis is descriptive and comparative and relies solely on publicly available global social and environmental datasets; no experimental treatments, interventions, or replicates were involved.
Research sample	This study did not involve biological samples, organisms, or human participants. Analyses were based on publicly available global datasets describing socioeconomic and demographic conditions and spatial layers representing potential conservation areas.
Sampling strategy	No sampling procedure was performed. The study is a global, spatially explicit analysis that incorporates all available data layers relevant to the scenarios examined; therefore, questions of statistical sampling do not apply.
Data collection	No new data were collected. All data were obtained from third-party, publicly available global datasets whose collection procedures are documented by the original providers.
Timing and spatial scale	All datasets represent the most recent global estimates available from their respective providers at the time of analysis. The study covers the entire terrestrial land surface (excluding Antarctica), with spatial resolution determined by each dataset and harmonised as needed for analysis. No temporal sampling or repeated collection was conducted.

Data exclusions | No data were excluded beyond the standard geographic masks applied consistently across all scenarios (e.g., removal of Antarctica or areas outside dataset coverage). No ad hoc exclusion criteria were applied.

Reproducibility | Reproducibility was ensured through scripted workflows implemented in R, the use of publicly available datasets, and the availability of the full analysis code in a public repository.

Randomization | Randomization was not applicable. The study did not involve allocation of subjects, organisms, or units to treatment groups; it is a global spatial analysis based on predetermined datasets.

Blinding | Blinding was not relevant to this study, as no experimental treatments, observations, or human participants were involved.

Did the study involve field work? ☐ Yes ☒ No

Reporting for specific materials, systems and methods

We require information from authors about some types of materials, experimental systems and methods used in many studies. Here, indicate whether each material, system or method listed is relevant to your study. If you are not sure if a list item applies to your research, read the appropriate section before selecting a response.

Materials & experimental systems

n/a	Involved in the study
☒	☐ Antibodies
☒	☐ Eukaryotic cell lines
☒	☐ Palaeontology and archaeology
☒	☐ Animals and other organisms
☒	☐ Clinical data
☒	☐ Dual use research of concern
☒	☐ Plants

Methods

n/a	Involved in the study
☒	☐ ChIP-seq
☒	☐ Flow cytometry
☒	☐ MRI-based neuroimaging

Plants

Seed stocks | No plant materials were used in this study. This field is not applicable.

Novel plant genotypes | No plant genotypes (transgenic, edited, hybridised or otherwise) were used or generated. This field is not applicable.

Authentication | No plant materials or genotypes were used; therefore, no authentication procedures were required. This field is not applicable.
